# The Role of Revefenacin in Chronic Obstructive Pulmonary Disease

**DOI:** 10.7759/cureus.4428

**Published:** 2019-04-10

**Authors:** Muhammad Haisum Maqsood, Kinza Rubab, Muhammad Arqam Maqsood

**Affiliations:** 1 Internal Medicine, King Edward Medical University / Mayo Hospital, Lahore, PAK; 2 Community Medicine, Combined Military Hospital Lahore Medical College, Faisalabad, PAK

**Keywords:** revefenacin, chronic obstructive pulmonary disease, copd

## Abstract

Chronic obstructive pulmonary disease (COPD) is a chronic inflammatory lung disease characterized by progressive and persistent airflow limitation that is not fully reversible. Revefenacin is an investigational long-acting muscarinic antagonist (LAMA), in late-stage development as a nebulized inhalation solution, which has been designed to produce long-acting bronchodilation, consistent with once-daily dosing, and with a high degree of lung-selectivity. It is more selective for muscarinic type 3 (M3) than muscarinic type 2 (M2) or muscarinic type 1 (M1) receptors. Its dissociation half-life for M3 is 82 minutes and 6.9 minutes for M1, respectively. The bronchoprotective effect is seen as early as five-minute post-dose and is sustained for up to 24 hours. The estimated 24-hour potency (expressed as the concentration of dosing solution) is 45.0 mg/ml. Once-daily dose of revefenacin provided long-term improvement in trough forced expiratory volume in one second (FEV1). It improved day 28 trough FEV1 over placebo significantly (*p* < 0.001). M3: M2 receptor half-life is 12 compared to the other antagonists that have M3: M2 receptor half-life around 6.0. A 24-hour serial spirometry, on day 84, showed that revefenacin 88 or 175 µg was associated with significant (*p *<0.01) improvements in trough FEV1 at all time points compared with placebo. Revefenacin is generally well-tolerated and unlike the other anti-muscarinics, it has no systemic anti-cholinergic adverse effects.

## Introduction and background

Description of the condition

Chronic obstructive pulmonary disease (COPD) is a chronic progressive inflammatory lung disease that is characterized by persistent yet partially irreversible airflow limitation [1-2]. Its symptomatic manifestations include dyspnea, chronic cough, and increased sputum production. COPD is currently one of the leading causes of death [3]. COPD is associated with a high morbidity and mortality rate and is currently a burden on society [4]. It has been estimated that by the year 2030, COPD will be the third leading cause of global mortality and will be directly responsible for 7.8% of all deaths [1]. Globally, chronic respiratory diseases are responsible for 6.3% of years lost due to disability, with COPD being the largest contributor. It makes up two-thirds of the global disability-adjusted life years [1].

Description of the treatment

To alleviate the symptoms, reduce the frequency and severity of exacerbations, and improve the health status are the goals of pharmacological therapy in COPD [5]. Long-acting inhaled bronchodilators are recommended as mainstay maintenance therapy for COPD in patients with moderate to severe symptoms [6]. These bronchodilators are categorized into two classes: long-acting muscarinic antagonists (LAMAs) and long-acting beta agonists (LABAs). Tiotropium is the once-daily LAMA, and the twice-daily LABAs include salmeterol and formoterol [7-9]. Muscarinic receptor antagonists inhibit hypersecretion of the mucous and relax the bronchial smooth muscles by reversing the cholinergic tone. They block the muscarinic type 1 receptor (M1) and muscarinic type 3 receptor (M3) [10-12]. The activation of the M2 autoreceptors, located in the pre-junction, inhibits excessive release of acetylcholine (ACh). Thus, blockade of M2 receptors increases ACh-mediated contractions [13-14]. Hence, anticholinergic drugs that preferentially antagonize muscarinic type 3 receptor (M3) should demonstrate improved efficacy compared with nonselective muscarinic receptor antagonists. Hand-held devices can result in inaccurate dosing, poor adherence and potentially poor clinical outcomes [15-16]. Nebulized therapy options for the maintenance treatment of COPD are currently limited [17].

Description of revefenacin

Revefenacin (TD-4208) is a LAMA that has been designed to produce long-acting bronchodilation, with once-daily dosing and with a high degree of lung-selectivity [18-20]. It is the first and currently the only once-daily, nebulized bronchodilator to be approved for the treatment of chronic obstructive pulmonary disease (COPD). On 9th November 2018, based on the results of three phase III trials, the US Food and Drug Administration approved revefenacin [21]. It prevents bronchoconstriction by inhibiting muscarinic M3 receptors in the airway smooth muscles. The approved dosage of revefenacin is 175 µg once daily and is delivered via a standard jet nebulizer connected to an air compressor [22]. After inhaled administration in COPD patients, revefenacin is rapidly metabolized in the liver to its major active metabolite (THRX-195518), with its plasma exposure being four to six-fold greater than revefenacin [23]. The potency of the active metabolite at muscarinic receptors is one-third to one-tenth lower than that of revefenacin [22]. Following repeated administration, steady state is reached within 7 days, with less than 1.6-fold accumulation. The mean steady-state volume of distribution is 218 L [22]. The elimination half-life of revefenacin and its active metabolite after once-daily dosing of inhaled revefenacin in COPD patients is 22-70 hours [22].

Why is it important to do this review?

There is limited data in the literature explaining the role of revefenacin in COPD. This has prompted us to search the existing literature and analyze the role of revefenacin in chronic obstructive pulmonary disease. In this article, we have highlighted the target and off-target effects of this novel drug as well as the pros and cons of it. This article has also compared revefenacin to the other existing anti-muscarinic agents. This comprehensive review of ours will benefit the medical professionals in knowing about better treatment options for COPD patients.

## Review

Lung selectivity

Revefenacin is a potent LAMA. It binds competitively and reversibly to the M3 receptors in the airway smooth muscle, causing bronchodilation. According to a study included in our review, the dissociation half-life was significantly (*p* < 0.05) longer for M3 than M2 receptors (82 minutes vs. 6.9 minutes). Its actions are site-specific [24]. Another study also showed that this novel long-acting bronchodilator, TD-4208, had greater lung selectivity with minimal systemic exposure. The study revealed that after seven days of repeat dosing in rats, salivation was inhibited to a much lesser extent than the other two muscarinic antagonists. Thus, it exhibits greater lung selectivity than either tiotropium or glycopyrronium [25]. One of the studies demonstrated that revefenacin dissociated significantly slower from the human muscarinic receptor 3 (half life = 82 minutes) compared to the muscarinic receptor 2 (half life = 6.9 minutes), making it kinetically selective for the M3 subtype [26]. 

Bronchoprotection

Revefenacin has sustained bronchoprotective effects [25]. According to a study, the bronchoprotective effect was seen as early as five minutes post-dose and was sustained for up to 24 hours [24]. The magnitude and duration of its bronchoprotective effect were similar to that of tiotropium and glycopyrronium. The estimated 24-hour potency was 45.0 mg/ml. The bronchoprotective potencies of TD-4208 and tiotropium were maintained after seven days of once-daily dosing, whereas glycopyrronium showed a six-fold loss in potency after repeat dosing. In dogs, the 24-hour in vivo bronchoprotective potency of TD-4208 was about 10-fold less potent than that of tiotropium. The difference in potency is consistent with the lower in vitro affinity of TD-4208 for human muscarinic M3 receptors relative to tiotropium [25].

Efficacy

One of the studies revealed that revefenacin significantly improved day 28 trough forced expiratory volume in one second (FEV1) over placebo (*p* < 0.001) [27]. Another study demonstrated that the 350μg dose did not demonstrate better efficacy than that observed with 175 μg of revefenacin. It has been suggested that 88 and 175 µg revefenacin are appropriate doses for the use in long-term safety and efficacy [27]. An efficacy analysis showed that inhaled revefenacin 88 or 175 µg once daily provided long-term improvement in trough forced expiratory volume in one second (FEV1) from baseline in patients with moderate to very severe COPD [24]. According to another study that was conducted on rats, revefenacin antagonized acetycholine-induced contraction of the airway with a potency (10.5) that was four-fold lower than that of tiotropium (11.1) but twenty-fold greater than that of ipratropium. Thus, their rank order potency is tiotropium > revefenacin = glycopyrrolate > ipratropium [26]. A study revealed that the mean peak FEV1 was significantly higher (*p* < 0.001) for revefenacin and ipratropium compared to placebo. FEV1 on day 7 was significantly higher (*p* < 0.006) for all revefenacin doses compared to placebo [28].

Duration of action

The literature demonstrated that TD-4208 has the potential to be a long-acting bronchodilator [25]. The antagonist effects of revefenacin and tiotropium persisted longer (half-life of 13.3 and >17 hours, respectively) compared with ipratropium (half-life = 1.6 hours). Revefenacin had the highest selectivity for the M3 receptor (M3: M2 receptor half-life= 12) compared to the other antagonists (M3: M2 receptor half-life = 6.6 and 6.0, respectively) [26]. The data has shown that the bronchodilator effect of revefenacin lasted more than 24 hours following nebulized administration [28].

Effect on forced expiratory volume in one second (FEV1)

The data in the literature revealed improvements in the 24-hour change from baseline in trough FEV1 [24]. According to a study that involved 24 hours serial spirometry, revefenacin was associated with significant (*p *< 0.01) improvements in trough FEV1 compared with placebo on day 84 [18]. On day 29, revefenacin and tiotropium improved trough FEV1 and forced vital capacity (FVC) from baseline, with improvements favoring revefenacin over tiotropium; though not significant (*p *= 0.104) [24].

Use of rescue medications

The data showed that the patients treated with revefenacin used less rescue medications throughout the treatment period. Doses ≥88 μg reduced the average number of albuterol puffs per day by more than one puff per day [27].

Off-target effects

The potency of revefenacin at the histamine H1 receptor and serotonin 5-HT4 receptor was >2700 fold and >1400-fold lower than at the M3 receptor. Thus, it implies that these interactions are unlikely to have clinical significance [26].

Adverse effects

Revefenacin was generally well tolerated and did not produce systemic effects typically associated with anti-cholinergic therapies [26-27]. It has been reported that the incidence of dry mouth was 1.4%, and it was lower than the 4% incidence resulting from tiotropium [25]. In another study, at least one adverse event was reported in patients receiving revefenacin 88 µg (53.4%) or 175 µg (50.7%) or placebo (48.3%) once daily; these were cough, headache, upper respiratory tract infection, nasopharyngitis and back pain [24]. AEs leading to treatment discontinuation occurred in 13% of revefenacin recipients and 19% of placebo recipients [24]. Following single-dose administrations in a study, the two most common AEs in all groups were headache (28.1%) and dyspnea (18.8%). No serious AEs leading to drug discontinuation were reported. No clinically significant changes in laboratory data or QT complex were observed [28]. All the important findings are summarized in Table 1, as shown below.

**Table 1:** Summary of important findings M1: muscarinic receptor type 1; M2: muscarinic receptor type 2; M3: muscarinic receptor type 3; FEV1: forced expiratory volume in one second

Feature	Summary	Reference number
Lung selectivity	Revefenacin has a high degree of lung selectivity. It is more selective for M3 than M2 or M1 receptors. Its dissociation half-life for M3 is 82 min and 6.9 min for M1.	[24, 25]
Bronchoprotection	The bronchoprotective effect is seen as early as five-minute post-dose and is sustained for up to 24 hours. The estimated 24-hour potency (expressed as concentration of dosing solution) is 45.0 mg/ml.	[25]
Efficacy	Once-daily dose of revefenacin provided long-term improvement in trough FEV1. It improved day 28 trough FEV1 over placebo significantly (p < 0.001).	[27]
Duration of action	Bronchodilator effect of revefenacin lasts more than 24 hours following nebulized administration. M3:M2 receptor half-life is 12 compared to the other antagonists which have M3:M2 receptor half-life around 6.0.	[26]
Effect on FEV1	24 h serial spirometry, on day 84 showed that revefenacin 88 or 175 µg were associated with significant (p< 0.01) improvements in trough FEV1 at all-time points compared with placebo.	[24]
Adverse effects	Revefenacin is generally well-tolerated and unlike the other anti-muscarinics, it has no systemic anti-cholinergic adverse effects.	[27]

## Conclusions

Revefenacin has the potential to be a long-acting bronchodilator for once-daily treatment of respiratory diseases. Its greater functional selectivity for the lung may translate to an improved tolerability profile compared with the other muscarinic receptor antagonists. Revefenacin could offer patients who require or prefer nebulized therapy the opportunity to be treated with a once-daily LAMA.
